# Institutional Questions

**Published:** 1900-08-18

**Authors:** 


### INSTITUTIONAL QUESTIONS.

### Schools of Tropical Medicine.

"A Correspondent" asks: Is the Liverpool School of Tropical Medicine in any way connected with that established at Greenwich, or is it a separate institution?

*** The School of Tropical Medicine at Greenwich is not connected with that at Liverpool. They are totally distinct and separate establishments. You may not have seen that recently the Government has been approached by the authorities of the Liverpool School, which is in connection with University College, Liverpool, with the request that it might be allowed similar recognition to that accorded to the London School. Lord Salisbury and Mr. Chamberlain, in granting the request, have stated that in future medical officers selected for service in any of the African States under the administration of the Government will receive a course of two months' instruction at one or other of these schools for the study of tropical diseases.

### Fire-places for Hospital Wards.

"Hospital Secretary" writes: My committee are contemplating putting new grates into the wards of our institution, and we should be glad if you would advise us as to the best form of fire-place to use. This is not a very new building.

*** You should consult your architect as to the form of grate most readily adaptable to the ward of your hospital. It is practically impossible to advise without acquaintance with many constructive details, which you do not give. Undoubtedly the "Teale" fire-places are the most desirable that can be procured, and you should write for catalogue to the Teale Fire-place Company, Leeds. These fire-places are in great request in hospital wards, and give much satisfaction in practical use. The "Well Fire" on somewhat the same principle is also a very good grate.

